# Molecular profiling and anti-infective potency of endophytic actinomycetes inhabiting *Madhuca insignis* Radlk., from Western Ghats of India

**DOI:** 10.1186/s43141-021-00135-0

**Published:** 2021-02-24

**Authors:** Soma Mondal, V. Ravishankar Rai

**Affiliations:** Department of Studies in Microbiology, University of Mysore, Manasagangotri, Mysuru, Karnataka 570006 India

**Keywords:** Endophytic actinomycetes, *Streptomyces*, *Madhuca insignis*, Drug discovery, Anti-microbial activity, TLC bioautography

## Abstract

**Background:**

Endophytic actinomycetes are well known for their diverse bioactive entities and considered as an important source for drug development research.

**Results:**

We isolated and identified four potential endophytic *Streptomyces* species, i.e., *Streptomyces misionensis* MI22, *Streptomyces roietensis* MI24, *Streptomyces glaucescens* MI29, and *Streptomyces* sp. MI04 inhabiting *Madhuca insignis* by its characteristic morphological features and 16S rRNA gene sequence analysis. *S. misionensis* MI22 exhibits a broad spectrum of anti-microbial activity against methicillin-resistant *Staphylococcus aureus* (25.00 ± 1.00 mm) followed by *Bacillus subtilis* (23.66 ± 0.57 mm), *Escherichia coli* (22.00 ± 0.00 mm), and *Candida albicans* (18.00 ± 0.00 mm). Minimum inhibitory concentrations of the ethyl acetate fraction of *S. misionensis* MI22 against test pathogens were ranged from 25 to 100 μg/mL. Indeed, strain MI22 also exhibited significant anti-proliferative activity against HeLa cell line with IC_50_ value 98 μg/mL and showed no cytotoxicity effect to the normal human embryonic kidney cell line in the MTT assay. The anti-microbial metabolites from strain MI22 were detected at *R*_*f*_ 0.55 as depicted by the inhibition zone on the intensive band in TLC-bioautography assay.

**Conclusion:**

The study indicates that, anti-microbial metabolites of these endophytic *Streptomyces* species, especially *S. misionensis* MI22 as a prolific source to discover novel bioactive metabolites to combat multidrug-resistant pathogens.

## Background

In the last few decades, more than 30 new infectious diseases have been emerged ranging from the rotavirus to Middle East respiratory syndrome coronavirus [1, 2]. The increasing people movement across diverse borders, the rapid expansion of air traffic and human population have modified the ecosystem has made these new pathogens can easily spread all over the globe. Therefore, the hunt for new potential drugs has always been essential throughout human history to combat infectious diseases.

Natural products or secondary metabolites originate from plants, bacteria, marine, and fungal sources are the most successful source of potent lead natural drugs [3]. They serve as a continuing potent source of novel bioactive agents, retaining an immense impact on modern medicine [4]. Antibiotic drugs like erythromycin, methicillin, and penicillin are used in a great extent to treat various diseases, but currently turning to be less potent as a result of many microbes become resistant to certain antibiotics [5]. The right solution to cover the problem of drug-resistant pathogens is to explore and discover new biomolecules.

Microbes are considered as a potent source of natural products as evidenced by the discovery of Penicillin from the fungus *Penicillium rubens* by Alexander Fleming in 1928 [6]. They have the ability to biosynthesize novel compounds that can be used for medical and agricultural applications [7, 8]. Indeed, plants with ethnomedicinal values are the potent sources of broad-spectrum bioactive agents. Microbial endophytes associated with medical plants play a vital role in secondary metabolism pathways and increase the ability to produce natural metabolites [9–11]. The endophytic actinomycetes constitute one of the fascinating groups of microbes associated with a wide range of medicinal plants. They are considered as a group of actinobacteria bearing the capacity to synthesize new bioactive secondary metabolites [12]. Biological diversity, species richness, and species distribution of endophytic actinomycetes are significantly influenced by ecological environs [13]. The endophytic actinomycetes diversity and anti-microbial activity inhabiting medicinal plants of the tropical region have been reported in the earlier studies [14–16], but the plants from Western Ghats region in India has not gained research attention.

The genus *Streptomyces* is known for a diverse array of bioactive agents like anti-cancer, anti-microbial, anti-infective biomolecules, and other pharmaceutical important drugs [17–21]. They are considered as prolific producers of diverse bioactive agents like antioxidants, antibiotics, enzyme inhibitors, and other biomolecules having therapeutic importance [22]. Most of the endophytic actinomycetes that have been exhibited anti-microbial potential belong to the *Streptomyces* genus, indicating that this particular genus is of great interest to discover novel anti-microbial drugs [23–25]. Hence, there is a need to explore actinomycetes diversity for bioactive metabolites.

*Madhuca insignis* is a riparian tree that belongs to the Sapotaceae family which is mainly distributed in Karnataka and Kerala states of South India [26–30]. It is a critically endangered and endemic riparian species found in the Western Ghats of India. Several reports are also documented on the pharmacological applications of *Madhuca* species [31]. There are no reports available in search of endophytic actinomycetes from *M. insignis*, despite its medicinal value. Hence, this study was designed to explore endophytic actinomycetes inhabiting *M. insignis* and to evaluate their anti-microbial and anti-proliferative potential against human pathogens.

## Methods

### Selection of plant and study site characteristics

Leaf, stem, and root samples of *M. insignis* were collected from Agumbe (13.5027° N, 75.0903° E), Western Ghats of India for the isolation of endophytic actinomycetes. The collected plant tissue samples were sealed using parafilm to keep its endophytic nature and brought to the laboratory within 24 h for the isolation of endophytes.

### Surface sterilization and isolation

Leaves, roots, and stem samples were first rinsed in running tap water and then with double-distilled water [25]. To get rid of surface microorganisms, samples were washed with 0.1% Tween 20 for 1 min and washed with 75% ethanol for 5 min. The samples were rinsed with 2% sodium hypochlorite for 5 min, and rinsed with 10% sodium bicarbonate for 5 min to inhibit the growth of fungi. Later, all samples were washed with double-distilled water and kept for surface drying. The plant tissue materials were cut into tiny segments (5 mm) and placed on starch casein agar (HiMedia, India) and incubated at 30 °C for 1 month. To confirm, the effect of the surface sterilization process, final wash aliquots of the sterile distilled was involuted on the culture plates. Petri plates were constantly checked for endophytic actinomycetes growth. The actinomycetes isolates were observed under the light microscope and scanning electron microscope. The morphological features like spiral sporophores, filamentous nature, hyphe, and Gram-straining were analyzed.

### Molecular identification of endophytic actinomycetes

#### Genomic DNA extraction, taxon sampling, and phylogenetic affiliation

To isolate genomic DNA, the endophytic actinomycete isolates were cultured in International *Streptomyces* Project-2 (ISP-2) broth for 7 days at 28 °C. DNA extraction was carried out following the standard protocol [32]. The amplification of the 16s rRNA gene was carried by a PCR method using universal primers, 27F (5′-AGAGTTTGATCMTGGCTCAG-3′) and 1492R (5′ TACGGYTACCTTGTTACGACTT-3′) [33]. PCR conditions: denaturation 95 °C for 5 min followed by 35 cycles at 95 °C for 1 min; annealing 54 °C for 1 min; extension 72 °C for 1 min, final extension at 72 °C for 10 min and cooling at 4 °C. The amplified PCR products were visualized in gel electrophoresis. 16S rRNA gene sequence was carried out in ABI 3730 Genetic Analyzer (Applied Biosystems, USA). The nucleotide sequences of endophytic actinomycetes with similar identities were retrieved from the National Center of Biotechnology Information (NCBI) database using BLAST search. The multiple sequence alignment was carried out using CLUSTAL Omega and the dendrogram was generated using MEGA 5.1 software [34].

### Extraction of secondary metabolites

The endophytic actinomycetes were cultured in 1 L Erlenmeyer flasks with 300 mL of ISP-4 broth and incubated at 28 °C for 1 month under static conditions. The mycelium and filtrate of culture broth were filtered to separate each other. The filtrate was blended thoroughly and centrifuged at 5000 rpm for 15 min. Ethyl acetate (v/v) was used to extract the liquid supernatant and evaporated to dryness at 45 °C using a rotary flash evaporator [35].

### Anti-microbial activity

Disc diffusion assay [36] was used to determine the anti-microbial activity of the ethyl acetate extract of endophytic actinomycetes against *Bacillus subtilis* (MTCC 121), Methicillin-resistant *Staphylococcus aureus* (ATCC 33915) and *Escherichia coli* (MTCC 7410), and *Candida albicans* (MTCC 183). Sterile discs were impregnated with 20 μL of extracts were placed on the medium seeded with test pathogens. Negative control of sterile disc with 20 μL of ethyl acetate was also impregnated for each test microbial pathogen with gentamicin (10 μg/disc) and nystatin (100 μnits/disc) as a positive control. Plates were then incubated for 24 h at 37 ± 2 °C and 28 ± 2 °C for bacteria and fungi, respectively [37]. The statistical analysis was carried out using IBM SPSS version (2018) software.

### Determination of minimum inhibitory concentration

The ethyl acetate extract of strain MI22 was evaluated for minimum inhibition concentration by 96-well plate method [34]. Test pathogens were cultured in sterile broth, and added to all well plates (10 μL each). The crude extract was tested in two-fold dilution (200–0.3906 μg/mL). Microbial growth indicators, 2,3,5-triphenyl tetrazolium chloride (for bacteria) and 3-(4,5-di-methylthiazol-2-yl)-2,5-diphenyl tetrazolium bromide (for fungi) were added to the each well. The plates were incubated for 37 ± 2 °C and 28 ± 2 °C for test bacteria and fungi, respectively. The lowest concentration of the extract with no microbial growth was determined as MIC.

### Thin-layer chromatography-bioautography

Anti-bacterial activity of strain MI22 was examined by thin-layer chromatography bioautographic method [38]. Ethyl acetate extract (10 μL) was spotted on the surface of TLC silica gel sheet and dipped in an optimum solvent system (petroleum ether/ethyl acetate 1:2). TLC chromatogram was dried and sterilized under UV light for 20 min. TLC chromatogram was carefully placed in a Petri plate containing brain heart infusion agar medium with 0.8% agar incorporated with TTC and MRSA pathogen. After 2 h of diffusion at 8 °C, Petri plate was incubated at 37 °C for 24 h.

### Anti-proliferative activity

Anti-proliferative activity of ethyl acetate fraction of strain MI22 was carried out on HeLa cell line by MTT assay [39]. Cytotoxicity of strain MI22 extract was also carried out on normal human embryonic kidney cell line (HEK 293T; a specific cell line originally derived from human embryonic kidney cells). Dulbecco’s modified eagle medium (DMEM) with 10% FBS, 100 mg/L penicillin, 250 mg L streptomycin, 2 mM glutamine was used to culture cells and incubated at 37 ± 2 °C with 5% CO_2_. Different concentrations of ethyl acetate fraction of strain MI22 (range 10 to 200 μg/mL) were added to wells. After 48 h, 10 μL of MTT (0.5 mg/mL) was added to all wells and incubated for 2 h at 37 ± 2 °C. The medium solution in PBS was removed, 100 μL of DMSO was added to all wells and 60 μM of curcumin (Sigma-Aldrich, India) was used as a positive control. The absorbance was determined by ELISA reader at 595 nm.

## Results

### Isolation of endophytic actinomycetes

**Fig. 1:**
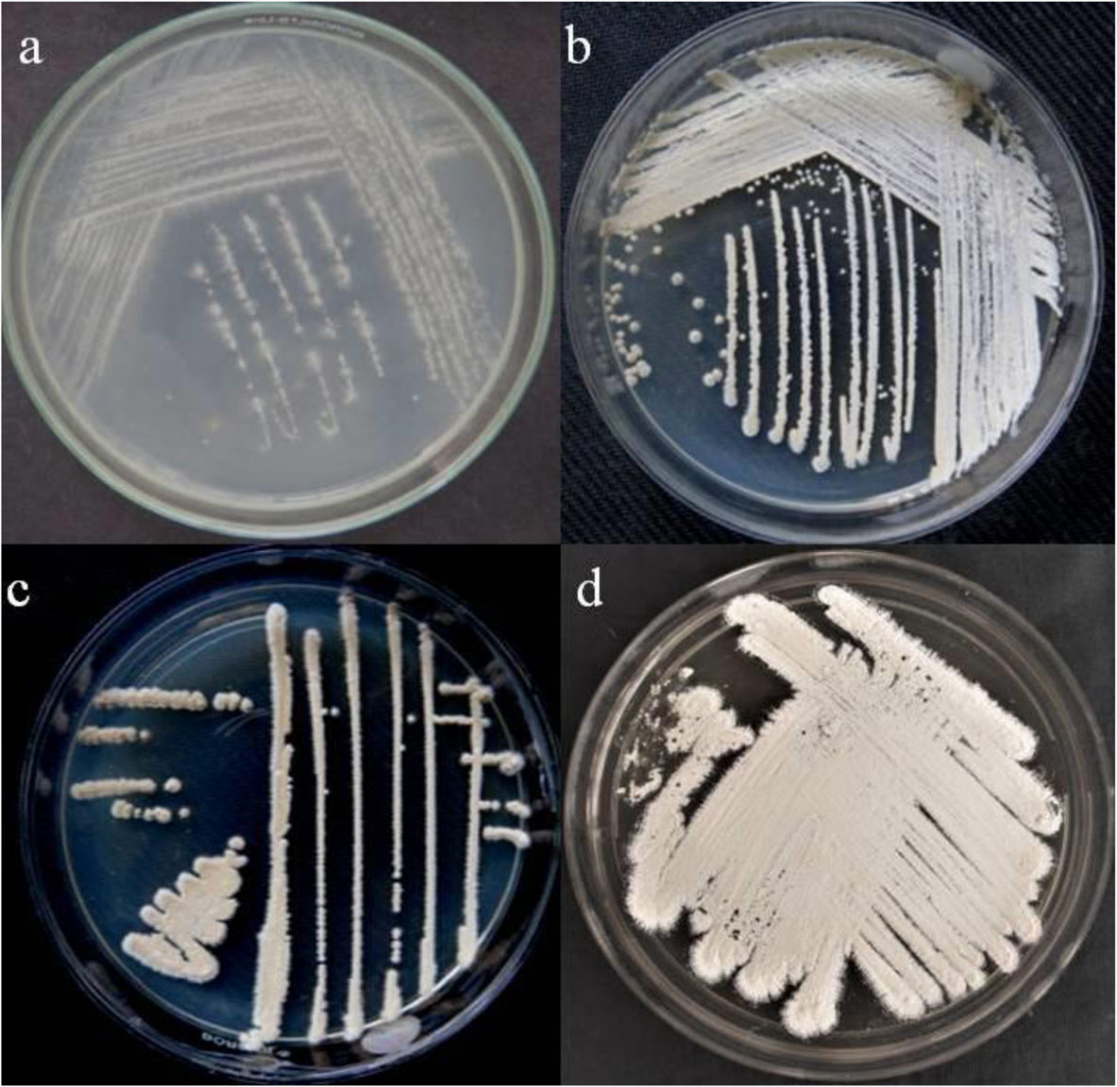
**a** *Streptomyces* sp. MI04. **b** *Streptomyces roietensis* MI24. **c** *Streptomyces misionensis* MI22. **d** *Streptomyces glaucescens* MI29

Isolation of endophytic actinomycetes was carried out from bark, root, and leaf tissues of *M. insignis*. The culture plates which showed actinomycetes growth were picked and four profusely grown endophytic actinomycetes were selected for further studies (Fig. 1). None of the colonies came out from the last wash of the sterilization process proving that the tissue surface sterilization was effective and isolates were true endophytes.

### Morphological, cultural, and phenotypic characteristics of endophytic actinomycetes

The morphological features of endophytic strains MI22, strain MI04, strain MI24, and strain MI29 cultured on actinomycetes isolation agar (AIA), starch casein agar (SCA), and International Streptomyces Project- 2 (ISP-2) media were analyzed. The growth pigment of all strains in the beginning appears to be whitish color in all media, however later differs with whitish powdery, creamy white and light brown colors. All selected strains exhibited melanin constitution which is a primary characteristic feature of *Streptomyces* sp.

The morphological characteristics of strain MI22 cultured on the SCA medium for 15 days were those typically exhibited by the genus *Streptomyces* (Fig. 1c). The strain MI22 showed typical morphology of *Streptomyces* with well-developed aerial mycelium and moderately grown substrate mycelium. The colonies had a convex structure, wavy periphery, and root striations. The fragmented mycelia formed straight spores with a smooth surface, light brown color as well as white powdery and smooth continuous spore ornamentation morphology.

**Fig. 2:**
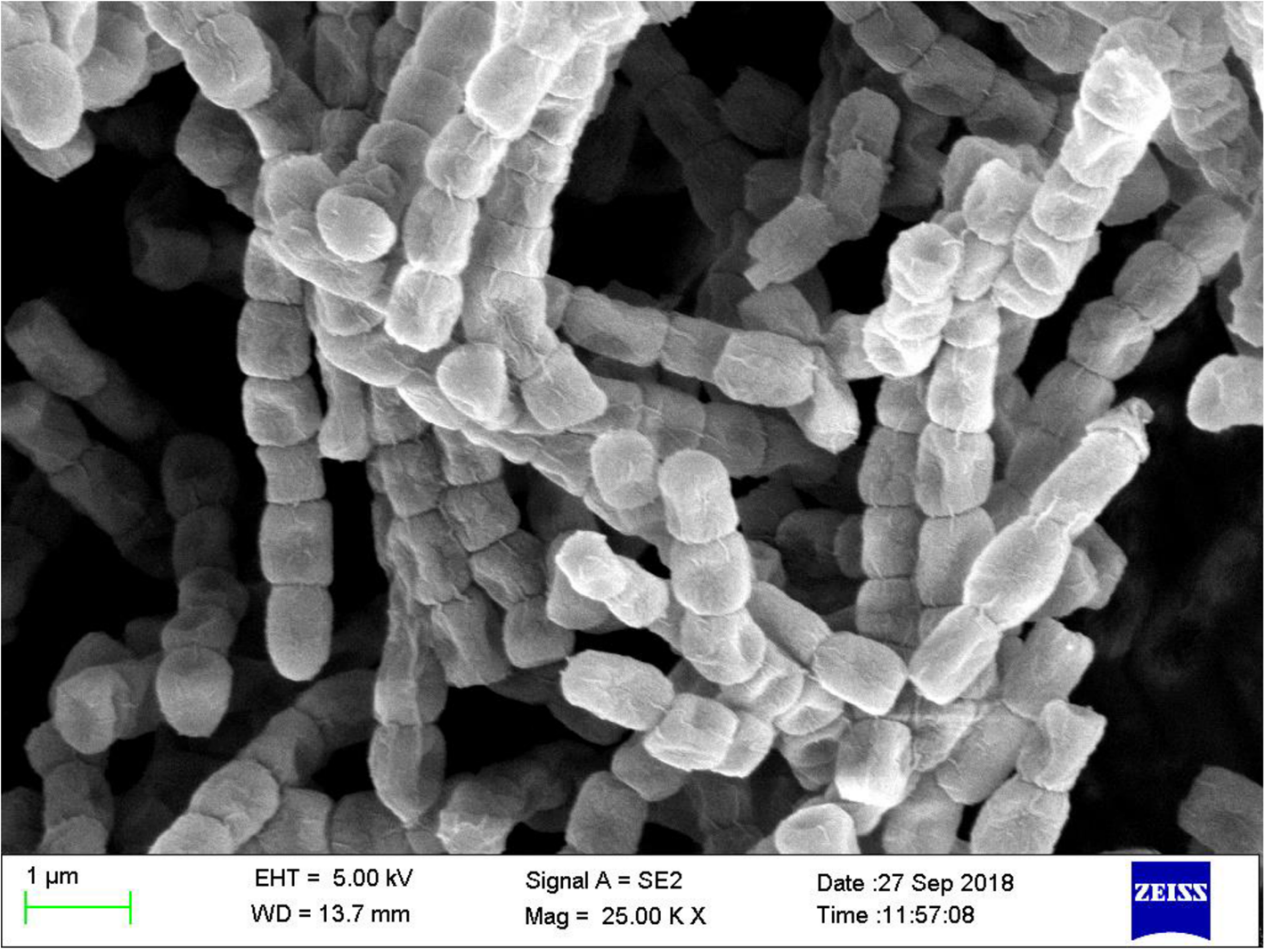
Scanning electron micrograph showing spore ornamentation in *Streptomyces* sp. MI04

The strain MI04 cultured on ISP-2 medium for 15 days were those typically exhibited by the genus *Streptomyces* (Fig. 2). The strain MI04 showed white brown rough colonies, spores-straight, wavy, or helical chain morphology characters with well-developed mycelium. The colonies are the convex structure with a wavy periphery and straight spores.

Morphological characteristics of the strain MI24 (Fig. 1b) showed *Streptomyces* morphology with well-developed substrate mycelium and aerial mycelium was formed moderately on SCA medium. The fragmented mycelium formed straight spores with a smooth surface, white powdery, and a light yellowish pigment was produced in nutrient agar medium.

The strain MI29 cultured on AIA medium colonies were white powdery, straight spores, wavy, or helical chains with a plain surface, light brown in color and smooth spore, continuous ornamentation morphology as *Streptomyces* sp. with matured aerial mycelium and moderately developed substrate mycelium (Fig. 1d).

### Molecular identification and phylogenetic affiliation

**Fig. 3:**
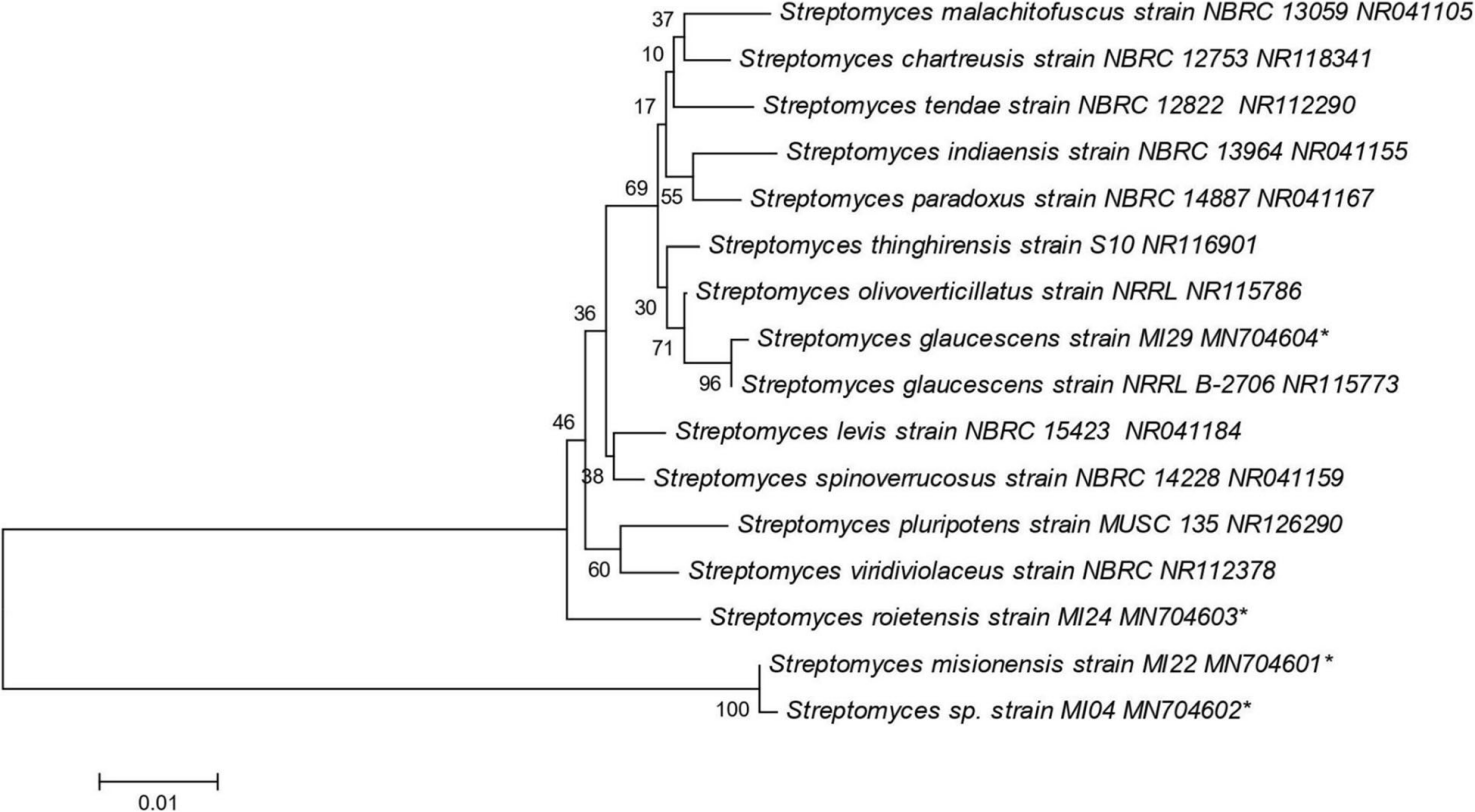
Phylogenetic tree retrieved from neighbor-joining analysis showing the evolutionary relationship of *Streptomyces misionensis* MI22, *Streptomyces* sp.MI04, *Streptomyces roietensis* MI24, and *Streptomyces glaucescens* MI29 with its closest BLAST hits. Bootstrap values (1000 replications) based on multiple sequence alignment using the MEGA software

To confirm the reliability of morphological identification, the endophytic actinomycetes strains were subjected to 16S rRNA gene sequence analysis. The obtained sequences were aligned with different strains of similar 16S rRNA sequences retrieved from the NCBI database. The consensus DNA sequences in BLASTN search and phylogenic analysis suggested that the isolates were identified as *Streptomyces misionensis* strain MI22, *Streptomyces roietensis* strain MI24, *Streptomyces glaucescens* strain MI29 and *Streptomyces* sp. strain MI04. The multiple sequence alignment that was performed using Clustal Omega retrieved from the NCBI database showed in several closely resembled sequences. A phylogenetic evaluation with related members of the genus *Streptomyces* was conducted and the results represented that our strains belong to the *Streptomyces* genus (Fig. 3). The DNA sequences of all these endophytic actinomycetes were deposited in GenBank and accession numbers were obtained (Table 1).

**Table 1:** List of endophytic actinomycetes strains isolated from *Madhuca insignis* with their morphological features and GenBank accession numbers

Plant	Plant part	Place of collection	Media used	Morphological characteristics	Name of the isolate	GenBank accession no.
*Madhuca* *insignis*	Stem	Agumbe (Mangalore)	SCA	White colony	*Streptomyces misionensis*MI22	MN704601
*Madhuca* *insignis*	Root	Agumbe (Mangalore)	ISP-2	Creamy Whitish colony	*Streptomyces* sp.MI04	MN704602
*Madhuca* *insignis*	Root	Agumbe (Mangalore)	SCA	Whitish powdery colony	*Streptomyces roietensis*MI24	MN704603
*Madhuca* *insignis*	Leaf	Agumbe (Mangalore)	Actinomycetes Isolation Agar	Whitish colony	*Streptomyces glaucescens*MI29	MN704604

### Anti-infective profiling

**Fig. 4:**
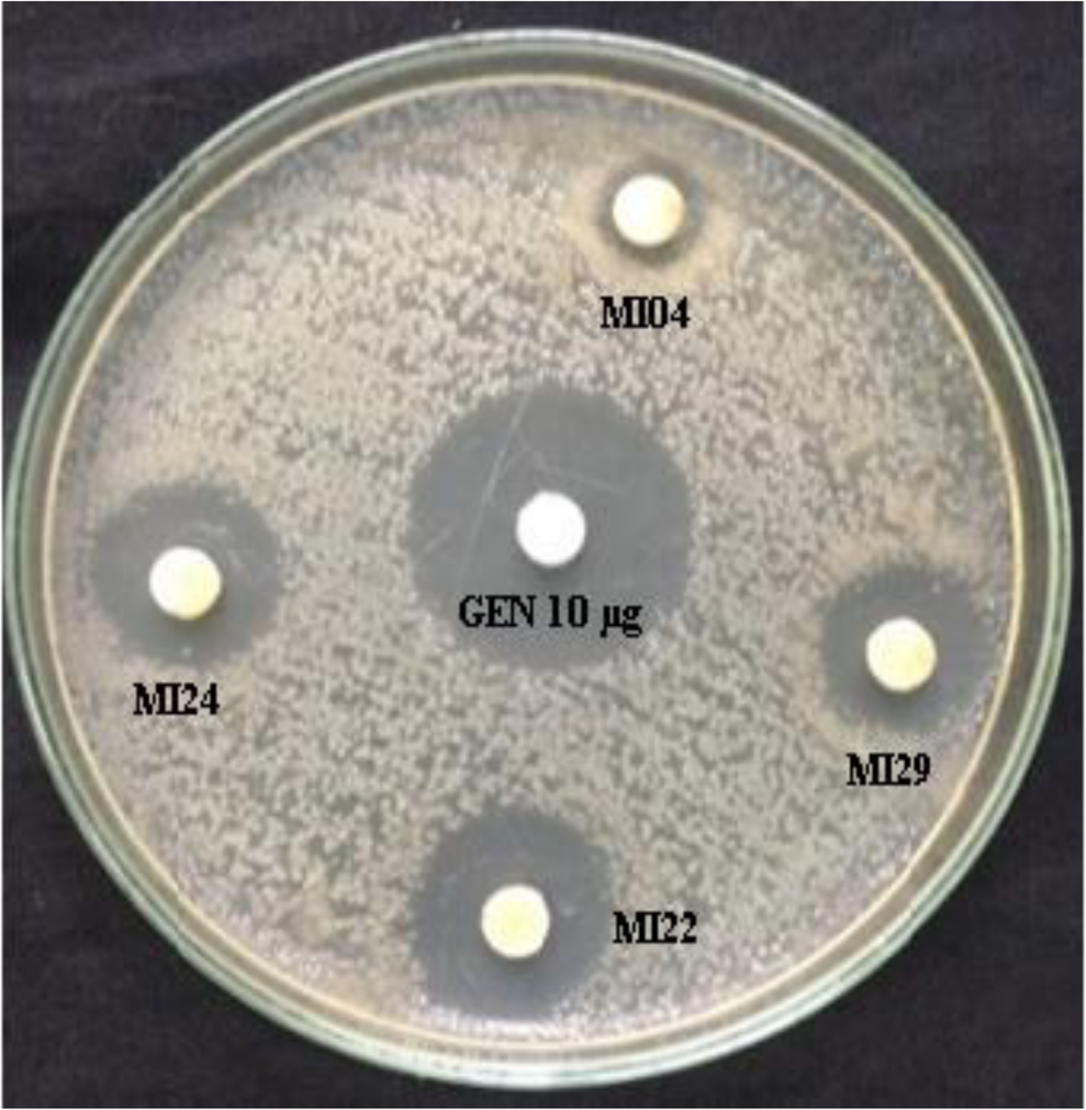
Anti-microbial activity of ethyl acetate extract of *Streptomyces* strains against Methicillin-resistant *Staphylococcus aureus* by disc diffusion assay

The anti-infective potential of ethyl acetate fractions of endophytic actinomycetes was determined by disc diffusion assay (Fig. 4). All endophytic actinomycetes strains showed anti-microbial activity against the test human pathogens (Table 2). *S. misionensis* MI22 exhibited broad-spectrum significant anti-microbial activity against all the tested pathogens followed by *S. glaucescens* MI29, *S. roietensis* MI24, and *Streptomyces* sp. MI04. *S. misionensis* MI22 exhibited significant anti-microbial potential which depicted inhibition zones against MRSA, (25.00 ± 1.00 mm), *Escherichia coli* (22.00 ± 0.00) *Bacillus subtilis* (23.66 ± 057 mm) and *Candida albicans* (18.00 ± 0.00 mm). The minimum inhibitory concentration test of ethyl acetate fractions showed potential anti-microbial activity ranging from 25 to 100 μg/mL. The anti-microbial potential of these endophytic actinomycetes were compared with standard antibiotics (Table 3).

**Table 2:** Determination of anti-microbial activity of ethyl acetate extract of endophytic *Streptomyces* strains against test microbial pathogens

Endophytic actinomycetes	Test pathogens			
	MRSA	***Bacillus subtilis***	***Escherichia coli***	***Candida albicans***
*Streptomyces misionensis*MI22	25.00 ± 1.00	23.66 ± 0.57	22.00 ± 0.00	18.00 ± 0.00
*Streptomyces* sp.MI04	12.33 ± 0.57	10.33 ± 0.57	19.00 ± 0.00	15.66 ± 0.57
*Streptomyces roietensis*MI24	21.00 ± 0.00	21.00 ± 0.00	21.33 ± 0.57	17.33 ± 0.57
*Streptomyces glaucescens*MI29	21.33 *± 0.57*	21.66 ± 0.57	24.33 ± 0.57	20.33 ± 0.57
Gentamicin (C)	29.33 ± 0.57	31.00 ± 0.00	30.00 ± 0.00	ND
Nystatin (C)	ND	ND	ND	25.00 ± 0.00

Value represents diameter of zone of inhibition in mm and data are means from 3 replicates ± SD. *ND* not determined, C-positive control, Gentamicin 10 μg/disc, Nystatin 100 units/disc

**Table 3:** Minimum inhibitory concentration of ethyl acetate *Streptomyces misionensis* MI22 against test human pathogens

Test pathogens	MIC in μg/ml
Methicillin resistance *Staphylococcus aureus*	25 μg/ml
*Bacillus subtilis*	25 μg/ml
*Escherichia coli*	50 μg/ml
*Candida albicans*	100 μg/ml

### Anti-proliferative activity

The HeLa cells were treated with different concentrations of ethyl acetate fraction of *S. misionensis* MI22 ranged from 10 to 200 μg/mL to obtain IC_50_ value. Ethyl acetate fraction of strain MI22 exhibited significant growth inhibitory efficacy on HeLa cell line with IC_50_ value 98 μg/mL. The cytotoxic potential of strain MI22 on the HEK293T cell line was turned to be negligible, indicating that crude extract was non-toxic to the non-cancerous cell. The positive control curcumin was active at 60 μM as the cells were reduced from 100%.

### Thin layer chromatography-bioautography

**Fig. 5:**
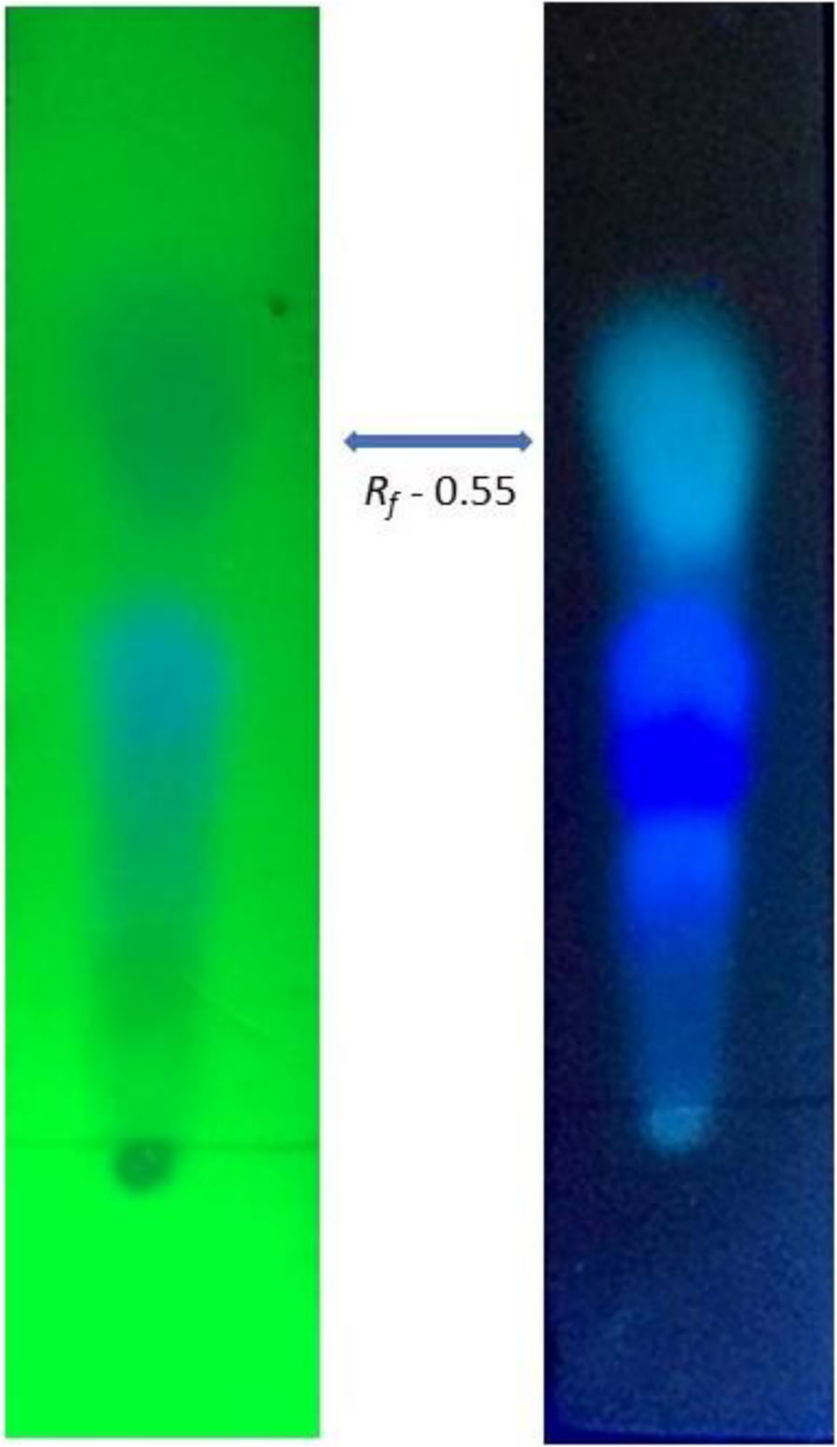
Thin-layer chromatogram of ethyl acetate extract of *Streptomyces misionensis* MI22 (**a** = 254 nm; **b** = 365 nm) showing an active band at *R*_*f*_ 0.55 responsible for anti-microbial activity

Ethyl acetate fraction of *S. misionensis* MI22 which showed anti-microbial activity was further tested in TLC-bioautography assay to detect the anti-microbial metabolites. TLC chromatographic profile showed the appearance of an intense band at *R*_*f*_ 0.55 under 254 and 365 nm (Fig. 5). The band appeared to be more intense which was due to the production of bioactive metabolites. A clear inhibition zone was observed on the same band in TLC bioautography where the medium was pre-inoculated with TTC agent and MRSA test pathogen.

## Discussion

Actinomycetes represent a large taxonomic group in the Kingdom of bacteria and are wide spread in nature [39, 40]. Their high metabolic diversity makes them great potential candidates for the discovery and development of new drugs [41]. In fact, new anti-microbial metabolites produced by medicinal plants are increasingly being discovered and are always challenged by fluctuations in their environment and are vulnerable to diverse endophytic microbes. Endophytic actinomycetes inhabiting the medicinal plants located in the Western Ghats of India are well known to biosynthesize new biomolecules and our research implies that the Western Ghats of India has a great diversity of endophytic actinomycetes [42].

During the present research isolation of endophytic actinomycetes, Petri plates spread with a final rinse of sterile surface water did not indicate the growth of any microorganisms. Thus, confirming that the surface sterilization method was effective to remove epiphytic microorganisms and therefore this protocol is the best method to acquire true endophytes. This research work constitutes the first report on the incidence of endophytic actinomycetes from the medicinal plant *Madhuca insignis.* Four endophytic actinomycete isolates were selected to explore their anti-infective potential.

Examination of the endophytic actinomycetes isolates using their spore chain, morphology, colony surface, spiral sporophores, hyphe, filamentous nature, and other characteristic features helped for the preliminary identification. Many of the morphological characteristics observed from our strains are the characteristics of the genus *Streptomycetes* [43–46]. In the present study, we describe the taxonomic position of isolates together with phylogenetic and morphological characteristics. The conventional method to identify actinomycetes is by using microscopes. However, for detailed observations of spore structures of *Streptomyces* strains, scanning electron microscope is required to differentiate *Streptomyces* species based on spore surface, spore chain morphology, the formation of single spores and spore surface textures [47, 48]. These morphological characters also have more significance in the modern era of *Streptomyces* taxonomy [49]. To support the reliability of morphological characterization, all strains were subjected to 16S rRNA gene sequence analysis.

Molecular identification using 16S rRNA gene sequences is a cost-effective tool to identify complex microbial strains at species level. The phylogenetic analysis was executed for all four isolates using 16s rRNA gene sequences to identify at species level. The amplified gene sequenced was aligned with closely related strains sequences that recovered from NCBI database determined by BLAST search. A phylogenetic evaluation with related members of the genus *Streptomyces* was conducted and the results represented that our member belongs to *Streptomyces* genus. The phylogenetic neighbor-joining analysis showed that ours belonged to the members of *Streptomyces misionensis* strain MI22, *Streptomyces roietensis* strain MI24, *Streptomyces glaucescens* strain MI29, and *Streptomyces* sp. strain MI04. Thus, our strains identified as *Streptomyces* species with strong support. Many phylogenetical unidentified endophytic actinomycetes exist which might have great implications in drug discovery and development research.

Anti-microbial susceptibility of all strains was performed to manifest the bioactive secondary metabolites in ethyl acetate fractions. Ethyl acetate was used to extract the bioactive metabolites as most of the secondary metabolites are miscible with it [50]. Among all strains, *S. misionensis* MI22 exhibits a broad spectrum of anti-microbial activities which suggest their ability as a potent anti-microbial agent. The capability to inhibit both fungal and bacterial growth suggests that secondary biomolecules contain a wide spectrum of anti-microbial agents. Various earlier reports have described that extracts of endophytic actinomycetes culture have several medicinal properties [25, 51–53]. The genus *Streptomyces* have been proven to have anti-microbial activity suggesting that this *Streptomyces* genus is of great significance for the biodiscovery and research development of novel anti-infective drugs [23, 24]. Our research investigation is similar to earlier reports from Wang et al. [54], Li et al. [55], Jiang et al. [56], and Sharma and Thakur [57] who state that *Streptomyces* species inhabiting host plants have significant anti-microbial activity. The hunt for a new drug with diverse biological activities has benefitted more attention in recent years [58]. In this viewpoint, these potential endophytic actinomycetes are a prolific source to discover novel secondary metabolites.

Anti-microbial TLC bioautography assay was used to monitor anti-microbial metabolite detection. *S. misionensis* MI22 showed the appearance of an intense band that appeared at *R*_*f*_ 0.55 confirmed the presence of anti-microbial agents [59]. Identification of anti-infective compounds through TLC-bioautography assay is one of the best, consistent and easy techniques to discover drugs from the natural origin [60]. Further studies are required to identify the secondary biomolecules to resist against multi-drug resistant microorganisms and human pathogens for anti-bacterial activity. Tumor cell lines were susceptible to the crude extract drug suggesting their broad-spectrum of the anti-cancer potential of *S. misionensis* MI22. The minimal cytotoxic capability on non-cancerous cell lines indicates the drug safe for the isolation of anti-cancer metabolites of pharmaceutical interest.

Based on these outputs, further biological studies like anti-malarial, antioxidant, and chemical profiling of these potent metabolites are crucial to develop potential drugs. Clearly, advanced studies on the formulation and new technologies are important to employ them in the medical and agricultural areas. Exploring these endophytic actinomycetes to find out natural ways to discover new bioactive metabolites are fascinating in drug discovery research. This is one of the important concerns in drug discovery to address the present difficulty to human health in the areas of several metabolic disorders. In view of all these, endophytic *S. misionensis* MI22 is a potential source for future novel antibiotics.

## Conclusion

This research indicates that targeting under-explored medicinal plants to explore hidden endophytic actinomycetes are a prolific source to discover new bioactive agents. *S. misionensis* MI22 as a potent source of bioactive drugs could help to fight against multidrug-resistant infections. Further studies on the purification, identification of bioactive metabolites from *S. misionensis* MI22 and their other biological including preclinical trials is under process. The overall results suggest that these potential endophytic *Streptomyces* strains could be used for the production of bioactive metabolites for pharmaceutical applications.

## Data Availability

All data generated or analyzed during this study are included in this published article.

## Abbreviations

NCBI: National Center of Biotechnology Information
ISP: International Streptomyces Project
PCR: Polymerase chain reaction
SCA: Starch casein agar
RF: Retention factor
DNA: Deoxyribonucleic acid
RNA: Ribonucleic acid
TTC: Triphenyl tetrazolium chloride
TLC: Thin layer chromatography
MTT: 1-(4,5-Dimethylthiazol-2-yl)-3,5-diphenylformazan
MIC: Minimal inhibitory concentration
MRSA: Methicillin-resistant *Staphylococcus aureus*
BLAST: Basic Local Alignment Search Tool
MTCC: Microbial type culture collection
ATCC: American Type Culture Collection
HEK: Human embryonic kidney
DMSO: Dimethyl sulfoxide
ELISA: Enzyme-linked immunosorbent assay
